# Former Road Cyclists Still Involved in Cycling Report Lower Burnout Levels Than Those Who Abandoned This Sport

**DOI:** 10.3389/fpsyg.2020.00400

**Published:** 2020-03-23

**Authors:** Fabrizio Sors, David Tomé Lourido, Stella Damonte, Ilaria Santoro, Alessandra Galmonte, Tiziano Agostini, Mauro Murgia

**Affiliations:** ^1^Department of Medicine, Surgery and Health Sciences, University of Trieste, Trieste, Italy; ^2^Department of Life Sciences, University of Trieste, Trieste, Italy; ^3^Department of Medical Area, University of Udine, Udine, Italy; ^4^Faculty of Psychology, National Distance Education University, Madrid, Spain

**Keywords:** burnout, cycling, sport abandonment, young athletes, emotional/physical exhaustion, reduced sense of accomplishment, sport devaluation

## Abstract

Despite the numerous benefits associated with sport practice, many children and adolescents end up quitting it year after year, with a stable dropout rate between 10 and 19 years of age. Among the causes of sport abandonment, the scientific literature highlights the presence of burnout as a fundamental factor. In this regard, the aim of the present study was to investigate the levels of the three components of sport burnout–emotional and physical exhaustion, reduced sense of accomplishment, and sport devaluation–reported by a sample of young (former) athletes, depending on whether their sport abandonment was relative (i.e., change to another sport modality) or definitive. In particular, participants were former agonist road cyclists, who have been divided into three groups on the basis of what they did after abandoning road bicycle racing, namely: (a) those still involved in cycling, either in a different specialty (e.g., mountain bike) or with a different role (e.g., coach for kids); (b) those who started practicing a different sport; and (c) those who definitively abandoned sports. The general hypothesis was that, with respect to those who changed sport and those who definitively abandoned it, those still involved in cycling would report experiencing lower levels of the three burnout components during the last year practicing it. To test this hypothesis, the Athlete Burnout Questionnaire (ABQ; Raedeke and Smith, 2001) was administered to 85 young former road cyclists. The results seem to support the hypothesis for two out of the three components, namely, emotional and physical exhaustion and sport devaluation; on the other hand, for reduced sense of accomplishment, no difference among the three groups emerged. Further research is needed to deepen the understanding of such processes, also in relation with other relevant constructs; yet, the results of the present study should already raise the awareness of sport organizations on the need to deal with this and related phenomena by adopting appropriate strategies to ensure the well-being of young athletes, thus trying to reduce early dropout.

## Introduction

The practice of physical activity and sports has been considered as a healthy habit for many decades (e.g., Tušak and Blatnik, 2014; Di Corrado, 2017; Petralia et al., 2018; Coco et al., 2019). Currently, there is consolidated evidence about how increasing exercise promotes self-esteem and cognitive functioning, as well as relieving depression levels in children and adolescents (Biddle et al., 2019). As long as the training load is appropriate for the maturation level of minors, it is unlikely that negative consequences for young athletes–such as injuries–can occur (McKay et al., 2019). However, despite the benefits undoubtedly associated with sport practice, many children and adolescents end up abandoning it year after year, with a stable dropout rate between 10 and 19 years of age (Møllerløkken et al., 2015). Crane and Temple (2015) conducted a systematic review on the issue of youth abandonment in organized sports, identifying five major areas related to it: lack of fun; perception of competence; social pressures; competitive priorities; and physical factors, such as maturation and injuries. Among the causes of sport abandonment, the scientific literature highlights the presence of burnout as a fundamental factor (e.g., Gustafsson et al., 2014; Isoard-Gautheur et al., 2016a; Larner et al., 2017).

The conceptualization of burnout arises in the clinical setting (Freudenberger, 1974). However, it is of great importance to contextualize it also in the sports environment; indeed, although most athletes do not experience this syndrome to a significant degree, it can lead to serious consequences for the physical and mental well-being of people who practice sports (Eklund and DeFreese, 2015). Burnout was defined in the sports context by Raedeke (1997), through a three-dimensional conceptualization, characterizing this syndrome by emotional and physical exhaustion, reduced sense of accomplishment, and sport devaluation. As a complement to this sports burnout model, and seeking to create a valid and reliable instrument, Raedeke and Smith (2001) developed the Athlete Burnout Questionnaire (ABQ) to measure the three postulated dimensions. This scale has shown good validity and reliability properties in different languages and cultural contexts (e.g., Arce et al., 2012).

In addition to the aforementioned sport abandonment, burnout syndrome has a series of negative consequences, such as depressed mood, psychological stress, or negative affect (De Francisco et al., 2016; Gustafsson et al., 2017a). The causes of burnout syndrome and the consequent abandonment of sports practice in young athletes are often related either with dispositional variables such as low levels of resilience and fear of failure (Gustafsson et al., 2017b; Sorkkila et al., 2019) or with motivational variables, linked to the non-satisfaction of the athlete’s basic psychological needs (Sorkkila et al., 2018; Lonsdale et al., 2009). Concerning the latter, according to the self-determination theory (Ryan and Deci, 2017), intrinsic motivation would be determined by the satisfaction of the psychological needs of autonomy, perceived competence, and relatedness with others. Such postulations have been corroborated in the sports context (Gustafsson et al., 2017a), with the observation that when athletes have a high intrinsic motivational profile, they have lower levels of burnout (Gustafsson et al., 2018a). In a complementary way, other researchers postulate that sport motivation is also determined by the achievement goal theory (Nicholls, 1984; Ames, 1995); in particular, intrinsic motivation would be linked to the task goals–those goals depending on the athlete’s effort–rather than to the ego goals, whose achievement is related to comparison with others in a range of superiority–inferiority (Lochbaum et al., 2016; Balaguer et al., 2017). The establishment of task-oriented goals, as well as of an empowering climate that favors athletes’ self-determination, is related to a lower presence of burnout (Lonsdale and Hodge, 2011; Vitali et al., 2014).

The fact that athletes are able to maintain a high intrinsic motivation–through the satisfaction of basic psychological needs and effort goals–depends also on other variables, such as family and/or coach support, which become crucial to prevent burnout (Isoard-Gautheur et al., 2016b; Ingrell et al., 2018). Focusing on the effects that these variables have on young athletes, it was found that those who receive greater social support from their team or have higher levels of sports competence and autonomy have lower levels of burnout and higher levels of intrinsic motivation (DeFreese and Smith, 2013; Rottensteiner et al., 2015; Jowett et al., 2016). On the other hand, the absence of fun or the establishment of ego goals is closely related to the presence of burnout symptoms, as well as to sport dropout (Gardner et al., 2017; Sorkkila et al., 2018).

To contribute to this line of research, the aim of the present study is to investigate the levels of the three components of sport burnout–emotional and physical exhaustion, reduced sense of accomplishment, and sport devaluation–reported by a sample of young (former) athletes, depending on whether their sport abandonment was relative (i.e., change to another sport modality) or definitive. Specifically, as concerns relative sport abandonment, we separately considered those who remained in the same sport–either in a different specialty or with a different role (e.g., as coaches for kids)–and those who started practicing a different sport. No previous studies investigated burnout with a similar approach; however, it is reasonable to put forward the general hypothesis that, with respect to those who changed sport and those who definitively abandoned it, those who remained in the same sport would report experiencing lower levels of the three burnout components during the last year practicing the specialty they then abandoned. As previous research highlighted a potential role of gender in quitting physical activity (e.g., Labbrozzi et al., 2012; Crane and Temple, 2015), we also tested for gender differences in reported burnout levels (regardless of the sport activity participants have undertaken after abandoning the specialty they practiced).

## Materials and Methods

### Participants

Eighty-five former agonist road cyclists (40 females, 45 males) were recruited as participants. At the moment of data collection, their age ranged between 18 and 25 years (M = 20.49, SD = 1.71). On average, they abandoned road bicycle racing when they were 16.35 years old (SD = 1.45); on average, the abandonment occurred 4.62 years (SD = 1.71) before data collection.

Participants were divided into three groups on the basis of what they did after abandoning road bicycle racing: (a) those still involved in cycling, either in a different specialty (e.g., mountain bike) or with a different role (e.g., coach for kids); (b) those who started practicing a different sport; and (c) those who definitively abandoned sports. From now on, the three groups will be called “Cycling other” (*N* = 33), “Different sport” (*N* = 29), and “No sport” (*N* = 23), respectively. Table 1 summarizes the descriptive data of the three groups; as can be noticed, the groups did not differ in terms of age at data collection, age at road bicycle racing abandonment, and years between abandonment and data collection.

**TABLE 1:** Descriptive data of the three groups of participants.

Group	Sex	Age at data collection (M ± SD)	Age at road bicycle racing abandonment (M ± SD)	Years between abandonment and data collection (M ± SD)
Cycling other	F = 12, M = 21	20.67 ± 1.65	16.64 ± 1.25	4.58 ± 1.46
Different sport	F = 15, M = 14	20.48 ± 2.05	16.00 ± 1.63	4.97 ± 1.95
No sport	F = 13, M = 10	20.26 ± 1.32	16.39 ± 1.44	4.26 ± 1.71

Informed consent was obtained from each participant prior to the beginning of the data collection. The protocol was approved by the Ethics Committee of the University of Trieste, and the study was carried out in accordance with its recommendations.

### Questionnaire and Procedure

The ABQ (Raedeke and Smith, 2001) is a self-report inventory consisting of 15 items, equally distributed among the three subscales of emotional and physical exhaustion, reduced sense of accomplishment, and sport devaluation. In the present study, the Italian translation used by Vitali et al. (2014) was administered. With respect to the original version, we modified the general request to refer to the last year participants practiced road bicycle racing, that is, “Thinking about your last year practicing road bicycle racing, rate how often you experienced the following situations”; accordingly, the tense of all items was changed from present to past. Items were to be answered like in the original version, that is, on a five-point Likert scale, with the following anchors: (1) almost never, (2) rarely, (3) sometimes, (4) frequently, and (5) almost always. For each participant, the score of each subscale corresponds to the average of the responses given to the five items characterizing it.

In addition, participants were asked to specify the following information: their sex, their current age, and their age when they abandoned road bicycle racing, the year they abandoned road bicycle racing, and what they did at a sport level after abandoning road bicycle racing.

For data collection, a digital survey was prepared by means of Google Forms, including both the ABQ and the personal questions mentioned above. Such a survey was preceded by the description of relevant information about the study, to allow participants to express their informed consent. The form was structured in such a way that when consent was expressed, the browser automatically redirected to the survey; on the other hand, when consent was denied, the browser automatically redirected to a standard exit page.

As concerns the procedure, in a preliminary phase, young former road cyclists from the Italian regions of Friuli Venezia Giulia and Veneto were contacted and informally asked for their availability to participate to the study. Those who agreed (>98%) were sent the link to the form described above.

### Statistical Analyses

Preliminarily, we calculated Cronbach’s alpha for each subscale of the ABQ. Then, to address the main research question of the study, a multivariate analysis of variance (MANOVA) was conducted, considering each of the three subscales of the ABQ in relation with what participants did after abandoning road bicycle racing; *post hoc* comparisons were run using the least significant difference (LSD) correction. Finally, to investigate for potential gender differences in reported burnout levels, another MANOVA was performed, considering each of the three subscales of the ABQ in relation with gender. The analyses were run using the statistical software SPSS 25.0 for Windows.

## Results

Cronbach’s alpha values were 0.87 for emotional/physical exhaustion, 0.81 for reduced sense of accomplishment, and 0.80 for sport devaluation.

With regard to the main research question, the MANOVA revealed a significant main effect for the subscales “emotional and physical exhaustion” [*F*(2,82) = 3.96; *p* < 0.05; η^2^ = 0.09; power = 0.70] and “sport devaluation” [*F*(2,82) = 4.16; *p* < 0.05; η^2^ = 0.09; power = 0.72]. Conversely, the analysis did not reveal a significant value for the main effect of the subscale “reduced sense of accomplishment” [*F*(2,82) = 0.11; *p* = 0.90].

As for emotional and physical exhaustion, the *post hoc* analyses highlighted: (a) a significant difference between the group *Cycling other* and the group *Different sport*, with lower levels for the former group (*p* < 0.05; *Cycling other*: M = 1.68, SD = 0.62; *Different sport*: M = 2.22, SD = 0.91); (b) a significant difference between the group *Cycling other* and the group *No sport*, with lower levels for the former group (*p* < 0.05; *No sport*: M = 2.11, SD = 0.89); and (c) no difference between the group *Different sport* and the group *No sport* (*p* = 0.88).

As for reduced sense of accomplishment, the *post hoc* analyses revealed no difference in the three comparisons between pairs of groups (*Cycling other* versus *Different sport*: *p* = 0.81; *Cycling other* versus *No sport*: *p* = 0.80; *Different sport* versus *No sport*: *p* = 0.64). In particular, the scores for each condition were the following: *Cycling other*: M = 3.28, SD = 0.80; *Different sport*: M = 3.23, SD = 0.84; and *No sport*: M = 3.34, SD = 0.94.

**FIGURE 1:**
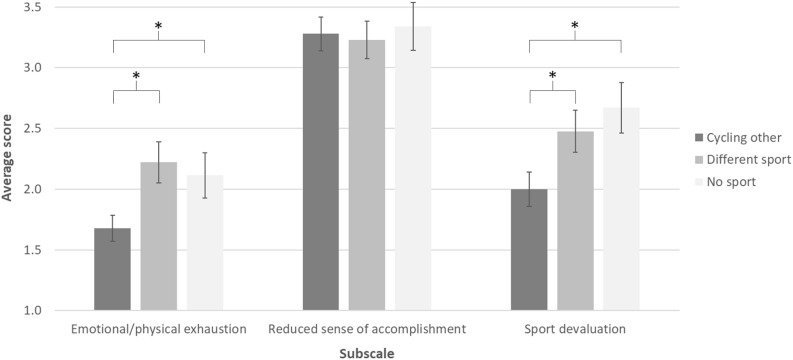
Average scores of the three groups for the three subscales of the Athlete Burnout Questionnaire (ABQ); error bars show the standard error of the mean; ^∗^*p* < 0.05.

As for sport devaluation, the *post hoc* analyses highlighted: (a) a significant difference between the group *Cycling other* and the group *Different sport*, with lower levels for the former group (*p* < 0.05; *Cycling other*: M = 2.00, SD = 0.82; *Different sport*: M = 2.48, SD = 0.93); (b) a significant difference between the group *Cycling other* and the group *No sport*, with lower levels for the former group (*p* < 0.05; *No sport*: M = 2.67, SD = 1.00); and (c) no difference between the group *Different sport* and the group *No sport* (*p* = 0.45). All results are summarized in Figure 1.

With regard to the potential gender differences in reported burnout levels, the MANOVA revealed no differences between females and males in any of the subscales (emotional/physical exhaustion: *p* = 0.21; reduced sense of accomplishment: *p* = 0.08; sport devaluation: *p* = 0.60). In particular, for emotional/physical exhaustion, the score for females was M = 1.86, SD = 0.75, while for males, M = 2.09, SD = 0.89; for reduced sense of accomplishment, the score for females was M = 3.11, SD = 0.94, while for males, M = 3.43, SD = 0.72; for sport devaluation, the score for females was M = 2.40, SD = 1.05, while for males, M = 2.29, SD = 0.84.

## Discussion

The aim of the present study was to investigate the levels of the three components of sport burnout–emotional and physical exhaustion, reduced sense of accomplishment, and sport devaluation–reported by a sample of young (former) athletes, depending on whether their sport abandonment was relative or definitive. The general hypothesis was that, with respect to those who changed sport and those who definitively abandoned it, those who remained in the same sport (in a different specialty or with a different role) would report experiencing lower levels of the three burnout components during the last year practicing the specialty they then abandoned. The results seem to support this hypothesis for two out of the three components, namely, emotional and physical exhaustion and sport devaluation. Indeed, the group *Cycling other*–that is, former agonist road cyclists still involved in cycling, either in a different specialty (e.g., mountain bike) or with a different role (e.g., coaches for kids)–reported experiencing lower levels of these two components during the last year practicing road bicycle racing with respect to both those who started practicing a different sport from cycling and those who definitively abandoned sports. Instead, for reduced sense of accomplishment, no difference among the three groups emerged. Interestingly, between the groups of those who changed sport and those who definitively abandoned it, no difference emerged also for the other two components. Moreover, as a secondary result, no gender differences emerged in any of the three burnout components.

There are two potential explanations for the main results. On the one hand, we can speculate that, during the last year practicing road cycling, participants had actually experienced the different burnout levels they have reported in the present study, and (also) based on these, they decided to remain in cycling or to abandon it. On the other hand, we can speculate that such differences did not actually exist, but the fact of remaining in cycling versus abandoning it has influenced participants’ memories about the burnout levels experienced during the last year practicing road cycling, with a softening effect for those who remained in cycling and a worsening effect for those who abandoned it. Of course, it is also possible–and reasonable–to think about a combination of these two explanations: some differences in burnout levels may have actually existed (contributing to the decision of remaining in cycling or not), and they could have been magnified by the subsequent experiences of participants.

As for the first potential explanation (i.e., the actual experience of different burnout levels), the establishment of a sport identity specifically associated with cycling might have occurred. The establishment of such an identity would be promoted by the satisfaction of the basic psychological needs in that sport in particular and by the resulting intrinsic motivation (Reifsteck et al., 2016). As children and adolescents play a particular sport, they develop an identity associated with their role as an athlete, as part of their self-concept (Brewer et al., 1993). However, if this identity is mono-dimensional–focusing only on sport practice and excluding other personal aspects–when sport abandonment occurs, it can lead to the suffering of psychiatric symptoms and burnout, both in young people and in adults (Giannone et al., 2017; Chang et al., 2018; Gustafsson et al., 2018b). As a consequence, especially for children and adolescents, on the one hand, it is essential to develop an identity as an athlete that allows them to feel competent and autonomous and maintain positive relationships with their peers; on the other hand, to prevent negative consequences in case of sport abandonment (either relative or definitive), such an identity should not be mono-dimensional, being preferably focused on their overall development as athletes rather than exclusively linked to their competence in a specific sport (Coakley, 1992). To evaluate the appropriateness of this interpretation, future studies aimed at investigating similar issues should take into consideration the potential mediating effects that the (non-)satisfaction of basic psychological needs could have on burnout levels. From a broader perspective, in future studies, former athletes from sports other than cycling should also be involved, in order to evaluate the degree of generalizability of the results that emerged from the present investigation. In particular, it would be interesting to see whether, when considering also former athletes from other sports, the absence of a difference concerning reduced sense of accomplishment, as well as the absence of a difference between those who changed sport and those who definitively abandoned it, is confirmed.

As for the second potential explanation (i.e., the influence of participants’ subsequent experiences on their memories), there are two aspects to be considered in this regard. On the one hand, it is well known that explicit memory is reconstructive, namely, people’s current thoughts and feelings influence the recall of their past thoughts and feelings (e.g., Levine, 1997; Ross and Newby-Clark, 1998); in particular, memories of emotional states are updated in light of subsequent experience (e.g., Safer et al., 2002; for a review, see Levine and Safer, 2002). On the other hand, people typically exaggerate the intensity of past emotions, overestimating the intensity of both the positive and negative emotions actually experienced (e.g., Wirtz et al., 2003; for a review, see Levine et al., 2006). In light of these two mechanisms–which are not mutually exclusive–and considering the fact that both those who remained in cycling and those who abandoned it had experienced negative emotions during their last year practicing road cycling, it is reasonable to hypothesize that all participants overestimated such negative emotions, but those still involved in cycling did it to a minor extent with respect to those who abandoned this sport. The experimental design used in the present study did not allow us to test this hypothesis; to evaluate its appropriateness, future studies on this topic should adopt a design typically used to investigate memories of emotional states, with repeated measurements during and after the event(s) of interest. However, identifying in advance athletes who are willing to abandon the sport they are practicing is not easy; a possible strategy is to start “monitoring” some teams toward the end of a sport season to identify the missing athletes at the beginning of the new one, so that the first measurement can occur only a couple of months after the abandonment (relative or definitive).

To sum up, the present study revealed that former agonist road cyclists still involved in cycling (either in a different specialty or with a different role) reported that they had experienced lower levels of emotional and physical exhaustion and sport devaluation during the last year practicing this specialty, with respect to both those who started practicing a different sport and those who definitively abandoned it. Interestingly, the presence of differences in these two burnout components, as well as the absence of differences in reduced sense of accomplishment, is coherent with a recent study by Gerber et al. (2019), which highlighted greater emotional/physical exhaustion and sport devaluation in young elite athletes reporting high compared to low burnout symptoms. A reasonable explanation for the results observed in our study is that some differences in burnout levels actually existed (contributing to the decision of remaining in cycling or not), and these differences have been magnified by the subsequent experiences of participants. Further research is needed to deepen the understanding of such processes, also in relation with other relevant constructs, like for example, the (non-)satisfaction of basic psychological needs. The secondary result of the absence of gender differences in the reported burnout levels suggests that burnout itself would not contribute to gender differences in the early dropout phenomenon.

Interestingly, the present study adds to the literature of psychology in the domain of cycling; previous psychological studies addressed other aspects related to cycling, such as mood (e.g., Murgia et al., 2016), recovery–stress balance (e.g., Filho et al., 2013, 2015), attentional focus (e.g., Bertollo et al., 2015; di Fronso et al., 2018), and psychobiosocial states (e.g., Robazza et al., 2017). As concerns psychobiosocial states, other studies highlighted their potential role as mediators of the relationship between the perceived motivational climate by young athletes and their motivation to continue playing sports (e.g., Bortoli et al., 2009, 2011, 2014); thus, psychobiosocial states also could be taken into consideration by future studies aiming to better understand the role of burnout in sport abandonment.

From an applied perspective, the results of the present study highlight two important aims to be pursued by sport organizations to prevent young athletes from experiencing negative emotions potentially leading to burnout and, consequently, to sport abandonment (relative or definitive): on the one hand, avoiding the exasperation of emotional and physical components of sport practice, and on the other hand, maintaining positive and meaningful feelings associated to it. It is quite intuitive that in this process, among all the figures populating sport organizations, a key role is played by the coaches; however, in dealing with these issues, young athletes may significantly benefit also from the interventions and the support provided by sport psychologists. From a broader perspective, findings deriving from studies on similar issues should raise the awareness of sport organizations on the need to deal with this and related phenomena by adopting appropriate strategies to ensure the well-being of young athletes, thus trying to reduce early dropout.

## Data Availability Statement

The raw data supporting the conclusions of this article will be made available by the authors, without undue reservation, to any qualified researcher.

## Ethics Statement

The studies involving human participants were reviewed and approved by the Ethics Committee of the University of Trieste. The patients/participants provided their written informed consent to participate in this study.

## Author Contributions

FS, IS, and MM conceived the idea. FS, DT, SD, IS, AG, TA, and MM designed the study. FS, SD, and AG prepared the digital form for data collection. SD recruited participants and collected the data. FS, MM, and TA analyzed the data. FS, DT, and MM wrote the first draft of the manuscript. SD, IS, AG, and TA revised the manuscript.

## Conflict of Interest

The authors declare that the research was conducted in the absence of any commercial or financial relationships that could be construed as a potential conflict of interest.
